# Sexual differences in locus coeruleus neurons and related behavior in C57BL/6J mice

**DOI:** 10.1186/s13293-023-00550-7

**Published:** 2023-09-28

**Authors:** Patricia Mariscal, Lidia Bravo, Meritxell Llorca-Torralba, Jone Razquin, Cristina Miguelez, Irene Suárez-Pereira, Esther Berrocoso

**Affiliations:** 1 Neuropsychopharmacology & Psychobiology Research Group, Department of Neuroscience, University of Cádiz, 11003 Cádiz, Spain; 2 Centro de Investigación Biomédica en Red en Salud Mental (CIBERSAM), Instituto de Salud Carlos III, 28029 Madrid, Spain; 3 Instituto de Investigación e Innovación Biomédica de Cádiz (INiBICA), Hospital Universitario Puerta del Mar, 11009 Cádiz, Spain; 4 Neuropsychopharmacology & Psychobiology Research Group, Department of Cell Biology & Histology, University of Cádiz, 11003 Cádiz, Spain; 5 Department of Pharmacology, Faculty of Medicine and Nursing, University of the Basque Country (UPV/EHU), 48940 Leioa, Spain; 6 Neurodegenerative Diseases Group, Biocruces Bizkaia Health Research Institute, 48940 Barakaldo, Spain

**Keywords:** Sex, Female, Locus coeruleus, Noradrenaline, Anxiety, Depression, Pain, Learning and memory, Electrophysiology, Patch clamp

## Abstract

**Background:**

In addition to social and cultural factors, sex differences in the central nervous system have a critical influence on behavior, although the neurobiology underlying these differences remains unclear. Interestingly, the Locus Coeruleus (LC), a noradrenergic nucleus that exhibits sexual dimorphism, integrates signals that are related to diverse activities, including emotions, cognition and pain. Therefore, we set-out to evaluate sex differences in behaviors related to LC nucleus, and subsequently, to assess the sex differences in LC morphology and function.

**Methods:**

Female and male C57BL/6J mice were studied to explore the role of the LC in anxiety, depressive-like behavior, well-being, pain, and learning and memory. We also explored the number of noradrenergic LC cells, their somatodendritic volume, as well as the electrophysiological properties of LC neurons in each sex.

**Results:**

While both male and female mice displayed similar depressive-like behavior, female mice exhibited more anxiety-related behaviors. Interestingly, females outperformed males in memory tasks that involved distinguishing objects with small differences and they also showed greater thermal pain sensitivity. Immunohistological analysis revealed that females had fewer noradrenergic cells yet they showed a larger dendritic volume than males. Patch clamp electrophysiology studies demonstrated that LC neurons in female mice had a lower capacitance and that they were more excitable than male LC neurons, albeit with similar action potential properties.

**Conclusions:**

Overall, this study provides new insights into the sex differences related to LC nucleus and associated behaviors, which may explain the heightened emotional arousal response observed in females.

**Supplementary Information:**

The online version contains supplementary material available at 10.1186/s13293-023-00550-7.

## Plain Language Summary

Exploring sex differences in the brain is important to understand the impact of such differences in pathological conditions characterized by gender bias, as well as their therapeutic implications. In this manuscript, we examined sex differences in the mouse locus coeruleus (LC) and how this might affect related behaviours. The LC is a sexually dimorphic nucleus that integrates signals associated with attention, anxiety, stress, arousal, pain, memory and learning. Our findings reveal that female mice exhibit more intense anxiety-related behaviors but that they perform better than males in recognizing small differences between objects. Additionally, we found pronounced sex differences in the LC, which contained fewer noradrenergic cells in females, with a larger dendritic volume and displaying enhanced cell excitability. These differences in the LC, a nucleus that fulfils a pivotal role in stress and pain, could be important for understanding the higher prevalence of stress-related disorders in women, such as anxiety and depression, but also of chronic pain. Hence, it is clearly important to consider sex differences in both preclinical and clinical research studies that attempt to understand pathologies related to these phenomena.

**Supplementary Information:**

The online version contains supplementary material available at 10.1186/s13293-023-00550-7.

## Highlights

- Female mice exhibit stronger anxiety-related behaviors than males, yet both show similar depressive-like behaviors.
- The discrimination index to distinguish similar objects with small differences is higher in females than in males.
- Females exhibit greater thermal pain sensitivity than males, yet both respond similarly to mechanical stimuli.
- Females have fewer noradrenergic cells in the LC yet they show a larger dendritic volume than males.
- LC neurons from female mice have a smaller cell capacitance but stronger excitability than those in males.

**Supplementary Information:**

The online version contains supplementary material available at 10.1186/s13293-023-00550-7.

## Introduction

It is known that sex differences at the level of the central nervous system can affect many biological activities and behaviors, such as stress-related responses, cognitive performance and pain [1–3]. Exploring sexual dimorphism in the brain is important to understand the impact of these differences, as well as their therapeutic implications in neurological and psychiatric diseases, given that gender bias is evident in several mental illnesses. Indeed, stress-related disorders like anxiety and depression, and chronic pain, are more common in women than in men [4–9]. Symptoms are also more severe in women with a younger age at onset, with prolonged or recurrent symptomatic periods and worse quality of life. Conversely, neurodevelopmental disorders like autism spectrum and attention-deficit disorders are more prevalent in boys than girls [10–12]. However, dissecting out the role of the biological and environmental influences for each disorder is challenging in humans. Therefore, preclinical studies in rodents, where a larger number of variables can be controlled, are adequate tools to investigate sexual dimorphisms in the brain and brain diseases. Such studies would encourage more research in the field and leading to the development of sex-specific and personalized diagnosis and treatment.

In recent decades, sexual dimorphism has been reported in several brain structures, such as the hypothalamus, hippocampus and locus coeruleus (LC) [13–17]. The LC is a brainstem noradrenergic nucleus that projects to a variety of regions through ascending and descending projections, and reciprocally, LC neurons receive extensive inputs from different brain regions [18]. Through these circuits, the LC integrates signals related to a variety of activities, including attention, anxiety, stress response, arousal/sleep, learning and memory, sensory processing, pain and reward processing [19–24]. In terms of LC sex differences, rat studies reported that the dendritic morphology of LC neurons is more complex in females than in males [25], with dendrites more prominent in the peri-LC region, and that the nucleus receives more stress-related afferents (e.g., from the central amygdala, the bed nucleus of the stria terminalis, etc.) [26]. However, these results are not always consistent due to variations in the methods and protocols used, or in the rat strains studied. In recent decades, more studies have been carried out on mice since the activity of specific neuronal subpopulations in the LC can be manipulated genetically, allowing the neural circuits that control certain behaviors to be modulated. In fact, transcriptional profiling of more than 3000 genes from the LC has revealed sex differences in more than 100 of these at the transcript level and different sex-related behavioral responses could be generated [27]. However, studies into the morphological and functional characteristics of LC neurons in naïve mice are scarce. Therefore, studying the sex differences in the LC nucleus in naïve animals, and their possible implications in anxiety and depressive-like behaviors, as well as pain thresholds and cognition, will be crucial to understand brain activity in a sex-specific manner.

Thus, this study was designed to investigate the differences in the LC of naïve male and female mice, and the implications of these in a variety of behaviors related to LC function, including anxiety, depressive-like behavior, well-being, learning and memory, and pain. The number of noradrenergic LC cells, the somatodendritic volume occupied and the electrophysiological properties of LC neurons were also evaluated to obtain a more comprehensive understanding of their function in C57BL/6J mice.

## Materials and methods

### Animals and experimental design

Male and female adult wild-type C57BL/6J mice (8–11 weeks of age) were maintained in separate rooms according to their sex under standard laboratory conditions (22 °C, 12-h light–dark cycle, and ad libitum access to food and water). All procedures were approved by the Committee for Animal Experimentation at the University of Cadiz and the UPV/EHU (M20/2021/234, Spain), conforming to the European Commission’s Directive (2010/63/EU) and Spanish Law (RD 53/2013) regulating animal research. Firstly, a behavioral evaluation was performed on three different sets of animals with n = 7–10 mice of each sex per set at 22 ± 1 °C and ∼ 13 lx of light. All the behavioral devices were cleaned with 70% ethanol between the testing of each animal, especially when changing between sexes. After performing the elevated plus maze (EPM) test, the estrous cycle of the female mice was assessed by vaginal cytology using crystal violet staining (Additional file 1: Fig. S1a) [28]. Immunohistochemistry studies were carried out after the behavioral assays. For that, 5 animals per group were evaluated using DAB (3,3′-diaminobenzidine tetrahydrochloride), and another 5 animals per group were assessed by immunofluorescence, analyzing the mean of the left and right LC for each animal. Moreover, the electrophysiological properties of LC neurons were analyzed in a different set of male and female mice (n = 5 per group).

### Emotional assessment

#### Elevated plus maze test

The EPM test was performed to evaluate anxiety-like behavior based on the tendency of rodents to explore novel environments, and their innate avoidance of unprotected and elevated places. An animal was placed in a gray cross-shaped maze (Panlab S.L., Barcelona, Spain) elevated 40 cm above the floor, with two open arms (29.5 cm × 6 cm) and two closed arms (29.5 cm × 6 cm × 15 cm-high walls), and with a central square (6 cm × 6 cm). The animal’s behavior was recorded over 5 min and the percentage of time spent in the open arms was determined using the SMART video-tracking software (Panlab, S.L., Barcelona, Spain), providing an estimate of anxiety-like behavior [29].

#### Light/dark test

The light/dark test evaluates anxiety-like behavior by assessing the animal’s displacement between two compartments of different sizes and colors, and with distinct illumination [30]. The apparatus consists of a small black compartment (25 cm × 16 cm) illuminated with a red bulb (~ 6 lx) and a larger white compartment (25 cm × 25 cm) lit with a white bulb (~ 1000 lx), the two separated by a connecting gate (7 cm × 7 cm) at floor level. Mice were placed into the dark compartment and allowed to move freely for 5 min. The latency to first enter the lit compartment, the total number of transitions (index of exploration) and the time spent in the lit compartment (reflection of aversion) was recorded automatically using weight transducer technology for animal detection, and with the PPCWIN software (Panlab, S.L., Barcelona, Spain).

#### Open field test

The open field test (OFT) was used to assess anxiety-like behavior as well as locomotor activity, as it is based on the rodent’s innate tendency to explore novel environments and avoid bright open spaces. The animal was placed in the center of a 45 cm × 45 cm square enclosure and allowed to move freely for 10 min while being recorded. The center was defined as a square area about 50% the size of the whole arena. The percentage of time spent in the central area and the total distance traveled were analyzed with the SMART video-tracking software (Panlab, S.L., Barcelona, Spain) [31].

#### Burrowing test

The burrowing test was performed to monitor animal well-being [32]. Mice were tested individually in a cage with a 154 mm long and 56 mm wide plastic tube filled with 140 g of food pellets. The burrow was located with the closed end against the back wall of the cage to provide sufficient distance for effective displacement of the burrowing material. Burrowing activity was calculated by subtracting the weight of the pellets present 1 h, 3 h, 6 h and 24 h after the start of the experiment from the original amount.

#### Sucrose splash test

The sucrose splash test consisted of spraying a 20% sucrose solution on the dorsal coat of the animal and recording its behavior was over the next 5 min. The grooming activity (licking, scratching and/or face-washing) was measured in seconds [33], whereby reduced grooming time indicated weaker motivational and self-care behavior.

#### Tail suspension test (TST)

The TST was used to evaluate depressive-like behavior. Mice were suspended from the distal end of the tail using adhesive tape and raised 20 cm above the floor during a 6 min recorded test session [34]. Their resulting behavior was analyzed to determine the time they spent immobile, defined as hanging by the tail without showing any active behavior. We also evaluated other behaviors [31], such as: (a) climbing—the mouse climbs up its tail; (b) swinging—the mouse moves its body from side to side; (c) curling—the mouse performs twisting body movements; and (d) clasping—the mouse retracts its hind limbs towards its abdomen.

#### Forced swimming test (FST)

The FST was used to evaluate depressive-like behavior [35, 36]. During a 6 min test, individual animals were placed in glass cylinders (10 cm diameter × 18 cm height) filled to a depth of 10 cm with water at 22 ± 1 °C. The sessions were recorded and the last 4 min were analyzed. Immobility behavior was determined when animals only undertook movements necessary to keep their head above water.

### Cognitive assessment

#### Novel object recognition (NOR) test

The NOR test relies on the innate preference of rodents for novelty. We used different NOR protocols to assess short-term (STM) and long-term memory (LTM), as well as a STM protocol to test the ability to discriminate small differences between objects. Mice were tested in a 45 cm × 45 cm square enclosure using different plastic objects (shapes, colors and textures). The mice were placed into the arena for a 10 min habituation phase in the absence of any objects, and then two identical objects were placed in opposite corners of the arena during a 15 min training phase. Subsequently, the mice were returned to their home cage for a delay period of either 1 h (STM) or 24 h (LTM). The mice were then subjected to a 10 min test in which one of the objects in the arena was replaced by a novel one, evaluating the animal’s object exploration activity defined as actively sniffing and/or touching the object while maintaining their gaze on the object. Circling or sitting on top of the object was not considered exploration. Object exploration was measured as the latency to the first object, the number of interactions, percentage preference and through a Discrimination Index (DI), the latter reflecting the amount of time spent exploring the novel object relative to the total time spent exploring both objects: DI = (Tnovel—Tfamiliar)/(Tnovel + Tfamiliar) [37].

### Sensory assessments

#### Manual von Frey test

Calibrated von Frey filaments (0.16, 0.40, 0.60, 1.0, 1.4, 2.0, 4.0, 6.0, 8.0 and 10 g: Bio-VF-M, BioSeb, France) were applied perpendicular to the plantar surface of each hind paw with just enough force to bend the filament. They were each applied 10 times to both paws, in ascending order, after a 30 min habituation in an individual plastic cage over a metal grid. The withdrawal response to the mechanical stimulus was considered as the rapid removal of the hind paw from the filament, usually followed by flinching or licking of the plantar surface. The percentage of response was derived from the number of withdrawals to each filament [38, 39].

#### Plantar test

Thermal thresholds were established through the Hargreaves’ method [40]. Mice were placed in individual plastic cages over an elevated glass surface and habituated for 45 min. Radiant heat was applied to the hind paw at a constant intensity using a Plantar test device (Ugo Basile, Italy), with a 30 s cut-off to prevent tissue damage. Two measurements were made and the mean latency of the paw withdrawal was considered as the thermal nociceptive threshold [41].

### Tissue processing, immunohistochemistry and immunofluorescence

#### Perfusion and sample extraction

At the end of the behavioral tests, 10 mice per group were anesthetized with 25% sodium pentobarbital and perfused transcardially through the ascending aorta with a 0.9% saline solution using a perfusion pump, followed by a 4% paraformaldehyde (PFA) solution prepared in 0.1 M phosphate buffered saline (PBS). The animal’s brain was removed carefully, post-fixed for an additional 2 h in 4% PFA, and then transferred to a 30% sucrose solution in phosphate buffer (0.1 M) with 0.1% sodium azide and left at least overnight at 4 °C. Coronal freezing microtome Sects. (40 µm) containing the LC were collected and stored in a cryoprotective solution at 4 ºC until further processing.

#### Structural and morphological studies

DAB immunostaining was performed on one of four series of 40 µm thick LC sections from 5 mice per sex. LC sections were probed for two nights at 4 °C with a primary antiserum against Tyrosine Hydroxylase (TH, rabbit anti-TH, 1:1000: OPA1-04050 Millipore), and then incubated with biotinylated donkey anti-rabbit antibodies (1:200: Jackson ImmunoResearch Europe, UK). Immunodetection was achieved using the ultra-sensitive ABC peroxidase staining kit (1:1000: Thermo Scientific, Spain) and DAB [42], and the sections were then mounted on slides, cleared in xylene and coverslipped with DPX. Images were acquired at the same exposure and illumination settings on an Olympus BX60 microscope equipped with an Olympus DP74 camera.

Immunofluorescence was performed as described previously [43] on all the 40 µm thick LC sections from 5 mice per sex. The same primary antiserum was used and revealed with the appropriate fluorophore-conjugated secondary antibodies (Donkey anti-rabbit Alexa 488, 1: 1,000: A-21206 Invitrogen). Sections were mounted onto glass slides with hard-setting antifade mounting media (Dako, S3023) and TH expression was captured at the same exposure from the rostral to caudal level of the LC (about − 5.32 mm to − 5.84 mm from Bregma) using a 20X oil immersion objective on a confocal microscope (Olympus FV1000).

To analyze the total number of LC neurons, TH^+^ neurons were counted manually using the ImageJ Cell Counter plugin, considering only those neurons whose nuclei could be visualized in the analysis, and the mean was calculated for the left and right LC of each animal. Cavalieri’s principle was used to obtain an unbiased stereological estimation of the LC volume [44], as V = ∑A x T, where ∑A is the sum of the areas measured in all LC sections and T is the distance between sections. The parallel LC images spaced 40 µm apart were stacked and analyzed using ImageJ (National Institutes of Health, Bethesda, Maryland). The area of staining (region of interest -ROI) was automatically outlined and measured in each section with the wand tool, using the 8-connected configuration mode to find connected regions. This protocol was performed by selecting the area occupied by the somas of the noradrenergic cells, as well as the area occupied by the entire LC by delimiting the somatodendritic area. The area occupied exclusively by the dendrites was obtained by subtracting the area occupied by the somas from the area of the entire LC. The mean was calculated for the left and right LC of each animal. Resulting areas were expressed as mm^2^ and the volumes as mm^3^.

### Electrophysiology

#### Slice preparation

Male and female mice (n = 5 per group) were sacrificed by decapitation under deep anesthesia (4% isoflurane), brains were removed and transferred to ice-cold artificial cerebrospinal fluid (ACSF, pH 7.4) equilibrated with 95% O_2_ and 5% CO_2_, and containing (in mM): 250 sucrose, 26 NaHCO_3_, 1.25 NaH_2_PO_4_.H_2_O, 0.5 CaCl_2_.2H_2_O, 10 MgSO_4_.7H_2_O, 10 D-glucose. Coronal sections of the brain containing the LC (220 µm thick) were obtained with a vibratome (VT1200S; Leica Microsystems, Germany) and slices were incubated in warmed (30–35 °C) ACSF for at least 30 min before recording, containing (in mM): 126 NaCl, 2.5 KCl, 1.25 NaH_2_PO_4_.H_2_O, 2 CaCl_2_.2H_2_O, 2 MgSO_4_.7H_2_O, 10 D-glucose, 26 NaHCO_3_, 1 sodium pyruvate and 4.9 L-glutathione [pH 7.4], gassed with 95% O_2_ and 5% CO_2_.

#### Whole-cell patch clamp recordings

Each slice was transferred to a recording chamber that was perfused continuously with oxygenated ACSF at 32–34 °C following our standard protocol [45, 46]. LC neurons were visualized using infrared gradient contrast video microscopy (Eclipse workstation, Nikon) and with a 60X water-immersion objective (Fluor 60X/1.00 W, Nikon). The LC was identified as a dense and compact group of cells at the lateral border of the central gray and the fourth ventricle, just anterior to the genu of the facial nucleus. Recordings from individual LC neurons were obtained with pipettes (impedance, 3–6 MΩ) prepared from borosilicate glass capillaries (G150-4: Warner Instruments, Hamdem, CR, USA). The patch pipette was filled with a KGluconate-based solution containing (in mM): 130 KGluconate, 5 NaCl, 1 MgCl_2_.6H_2_O, 10 HEPES, 1 Na_4_EGTA, 2 MgATP, 0.5 NaGTP, and 10 Na_2_PCr. The junction potential between the electrode solution and the external media (empirically estimated as 13 mV) was not corrected, and electrode signals were low-pass filtered at 4 kHz and sampled at 20 kHz. LC neurons were identified by the presence of a resting inwardly-rectifying potassium (IRK) conductance by stepping the membrane potential from − 40 to − 120 mV in − 10 mV increments (100 ms/step) [47]. In voltage clamp experiments, neurons were maintained at − 60 mV and the series resistance was monitored with steps of − 5 mV at the end of each recording. Data were discarded when the series resistance increased by > 20%. The average current response was analyzed off-line and the cell capacitance (Cm) and membrane resistance (Rm) were calculated. In the current clamp mode, incremental currents from − 150 to + 300 pA were injected in 25 pA steps to explore the subthreshold and firing properties of the neurons. Off-line analysis was performed using pClamp V9.2 (Molecular Devices, San Jose, CA, USA).

### Statistical analysis

All the data are presented as the mean ± SEM and the statistical analyses were performed using GraphPad Prism software (GraphPad Software 9.0.3, La Jolla, CA). Grubbs’ test was used to identify any statistical outliers and normal distributions of the data was confirmed with the Shapiro–Wilk test. Differences between two groups were determined using unpaired Student *t*-tests (two-tailed) when normally distributed or the non-parametric Mann–Whitney U tests when not. For comparisons between the soma, somatodendritic and dendritic distributions, as well as in the burrowing test, the data from each group was analyzed through the area under the curve (AUC). The Chi-squared test was used to analyze frequency distributions. Differences between more than two groups were determined using one-way ANOVA followed by a Tukey post hoc test. The material burrowed along hours, the percentage of response to the mechanical stimuli, as well as the IRK currents, firing frequency and voltage response to current injections were analyzed using repeated-measures ANOVA followed by Tukey post hoc test. Significance was set at p < 0.05.

## Results

### Female mice exhibit more anxiety-related behaviors than male mice

**Fig. 1:**
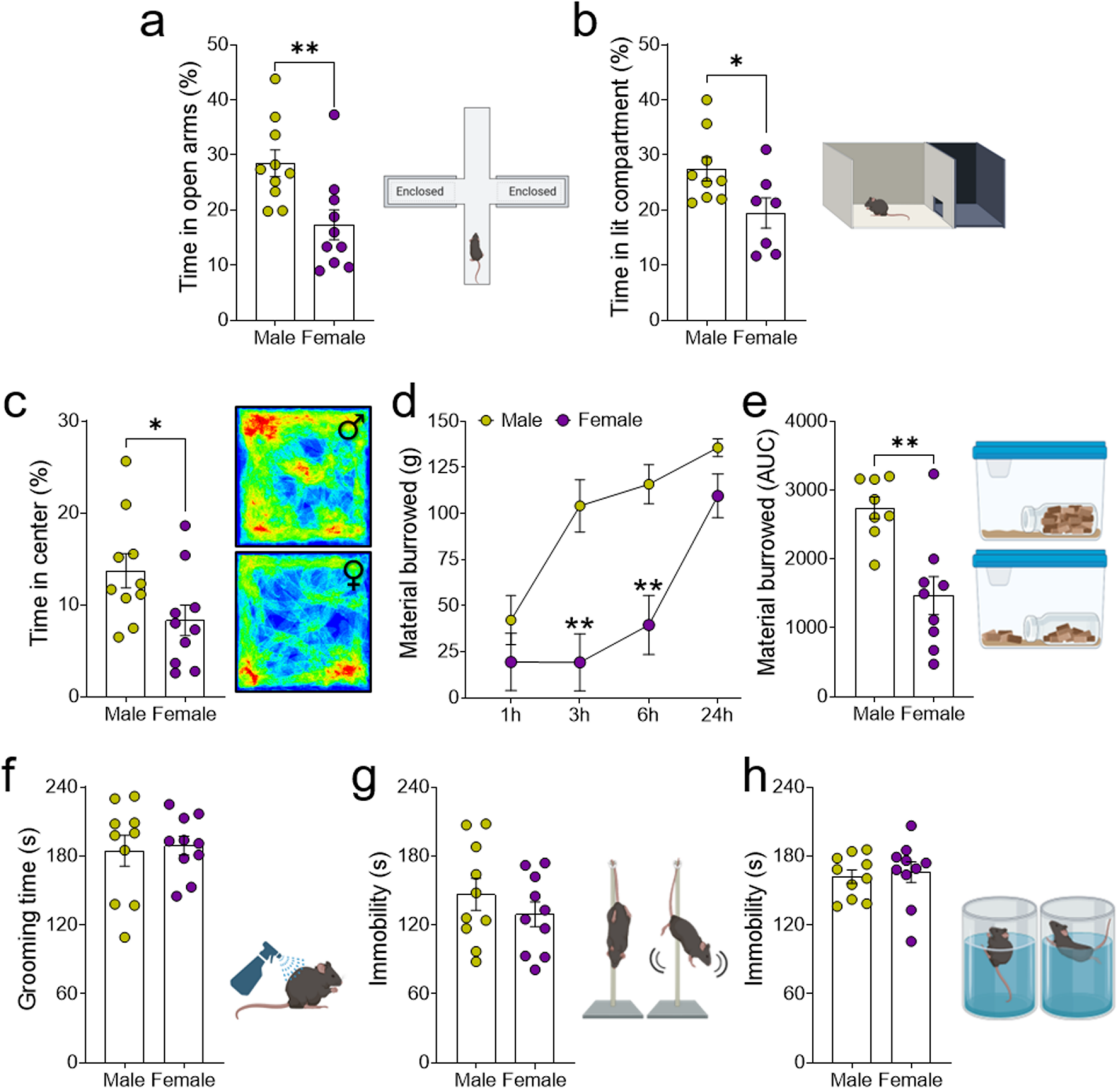
Evaluation of anxiety, well-being and depressive-like behavior in male and female mice. The results of anxiety-like behavior expressed as **a** the relative time spent in the open arms of the EPM by male and female mice. **b** Percentage of time spent in the lit compartment in the light–dark test. **c** Percentage of time spent in the central zone in the OFT, along with representative heatmaps of activity. **d** Graph depicting the material burrowed (in grams) and **e** the AUC of the material burrowed over time. **f** Time spent grooming in the splash test. Evaluation of the depressive-like behavior expressed as **g** immobility time in the TST and **h** FST. The data are presented as the mean ± SEM of n = 7–10 mice per group: *p < 0.05, **p < 0.01 vs male. From **b** to **h** females were in proestrus and estrus (P/E) stages. AUC, area under the curve

Female mice spent significantly less percentage of time in the open arms of the EPM (p < 0.01: see Table 1 and Fig. 1a), and a subsequent exploration of the estrous cycle revealed that female mice in the proestrus and estrus (P/E) stages spent less time in the open arms than those in the diestrus and metestrus (D/M) stages (p < 0.05: Additional file 1: Fig. S1b). Indeed, in these latter two stages the percentage of time spent in the open arms was similar to that of males. Furthermore, similar values of total activity (Additional file 1: Fig. S1c) and a similar number of entries into the open arms were observed (Additional file 1: Fig. S1d), suggesting a comparable level of exploration between sexes. Thus, the following behavioral tests were performed on females in the P/E stages. Similar results were found in the light/dark test, where females spent less percentage of time in the lit compartment than males (p < 0.05: Fig. 1b). However, the latency of both sexes to enter the dark compartment was similar (Additional file 1: Fig. S1e), as was the number of transitions between compartments (Additional file 1: Fig. S1f), suggesting a similar index of exploration in both groups. More anxiogenic behavior in the OFT was also attributed to female mice, which spent less percentage of time in the center of the arena than males (p < 0.05: Fig. 1c) even though the total distance traveled was similar in both these groups (Additional file 1: Fig. S1g).

**Table 1:** Summary of statistical analysis of LC-related anxiety and depressive-like behaviors

Shapiro–Wilk normality test (W, P value, Passed normality test?)										
	Male				Female					
Time in open arms (%)	0.9234 0.3865 Yes				*All* 0.8643 0.0857 Yes		*P/E* 0.9315 0.5639 Yes		*D/M* 0.8406 0.2155 Yes	
Time in lit compartment (%)	0.8558 0.0864 Yes				0.9143 0.4264 Yes					
Time in center (%)	0.9206 0.3617 Yes				0.9046 0.2456 Yes					
Material burrowed (g)	*1 h* 0.9296 0.5120 Yes	*3 h* 0.8623 0.1266 Yes	*6 h* 0.8313 0.0613 Yes	*24 h* 0.4623 < 0.0001 No	*1 h* 0.5049 < 0.0001 No	*3 h* 0.4993 < 0.0001 No		*6 h* 0.8261 0.0404 No		*24 h* 0.8238 0.0381 No
AUC (Material burrowed)	0.9049 0.3197 Yes				0.9196 0.3887 Yes					
Grooming time (s)	0.8880 0.1611 Yes				0.9472 0.6354 Yes					
TST Immobility time (s)	0.9268 0.4172 Yes				0.9310 0.4580 Yes					
FST Immobility time (s)	0.9173 0.3349 Yes				0.8900 0.1698 Yes					
EPM Total activity (AU)	0.9425 0.5806 Yes				0.9622 0.8109 Yes					
Entries in open arms	0.9464 0.6265 Yes				0.9103 0.2833 Yes					
Latency to dark compartment (s)	0.8811 0.1612 Yes				0.8739 0.2005 Yes					
Number of transitions	0.9165 0.3644 Yes				0.9380 0.6209 Yes					
OF Total distance (AU)	0.8886 0.1636 Yes				0.9109 0.2872 Yes					
Latency to groom (s)	0.9813 0.9717 Yes				0.8561 0.0686 Yes					
Latency to immobility (s)	0.8723 0.1063 Yes				0.9226 0.3793 Yes					

Unpaired Student’s t-test (t_(df)_)								
Time in open arms (%)	**t**_**(18)**_ = 3.06**							
Time in lit compartment (%)	**t**_**(14)**_ = 2.32*							
Time in center (%)	**t**_**(18)**_ = 2.16*							
AUC (Material burrowed)	**t**_**(15)**_ = 3.86**							
Grooming time (s)	t_(18)_ = 0.28							
TST Immobility time (s)	t_(18)_ = 0.99							
FST Immobility time (s)	t_(18)_ = 0.36							
EPM Total activity (AU)	t_(18)_ = 1.99							
Entries in open arms	t_(18)_ = 0.34							
Latency to dark compartment (s)	t_(14)_ = 0.07							
Number of transitions	t_(14)_ = 0.55							
OF Total distance (AU)	t_(18)_ = 0.85							
Latency to groom (s)	**t**_**(18)**_ = 3.69**							
Latency to immobility (s)	t_(18)_ = 0.46							

One-way ANOVA (F_(df. residual)_)								
Time in open arms by estrous cycle (%)	**F**_**(2,17)**_ = 12.32***							

Repeated-measures ANOVA (F_(df. residual)_)						
	Sex	Time	Sex × Time			
Material burrowed (g)	**F**_**(1, 15)**_ = 11.08**	**F**_**(3, 45)**_ = 39.14***	**F**_**(3, 45)**_ = 7.12***			

Chi-square test (*χ*^2^_(df)_)								
Clasping behavior (%)	*χ*^*2*^_(1)_ = 0.00							
Curling behavior (%)	*χ*^*2*^_(1)_ = 2.67							
Swinging behavior (%)	*χ*^*2*^_(1)_ = 0.00							
Climbing behavior (%)	*χ*^***2***^_**(1)**_ = 9.52**							

P/E, Proestrus/Estrus; D/M, Diestrus/Metestrus; AUC, area under the curve; TST, tail suspension test, FST, forced swimming test; EPM, elevated plus maze; OF, open field; AU, arbitray units; df, degrees of freedom

*p < 0.05; **p < 0.01; ***p < 0.001

Values in bold indicate statistically significant results

The burrowing test was used to evaluate the general well-being of the animals. It is based on the natural instinct of rodents to burrow, and the test involves placing a rodent in a cage with a burrow (a container filled with substrate like food pellets) and allowing them to burrow overnight. The amount of material displaced from the burrow was measured to quantify the burrowing behavior at different time points [32]. Our results showed that females were less apt to remove pellets from the burrow than males 3 and 6 h after the beginning of the test (p < 0.01: Fig. 1d). Accordingly, the AUC analysis revealed that females displaced significantly less material from the burrow than males (p < 0.01: Fig. 1e). In the splash test, the latency to grooming of females was lower than that of males (p < 0.01: Additional file 1: Fig. S1h), although the total grooming time was similar in both groups (Fig. 1f).

Behavioral despair was evaluated using the TST and FST. In the TST, there was no significant difference between the groups in the time spent immobile (Fig. 1g) but when analyzing other behaviors adopted during the test, the Chi-squared analysis revealed that the proportion of females that climbed was significantly higher than that of males (p < 0.01: Additional file 1: Fig. S1i). However, the test failed to detect differences between the sexes in swinging, curling or clasping behaviors (Additional file 1: Fig. S1i). Similar results were obtained in the FST, where mice showed no differences in the immobility time over the last 4 min of the test (Fig. 1h), or in the latency to immobility (Additional file 1: Fig. S1j).

### Sex differences in novel object recognition depend on the ability to distinguish details

**Fig. 2:**
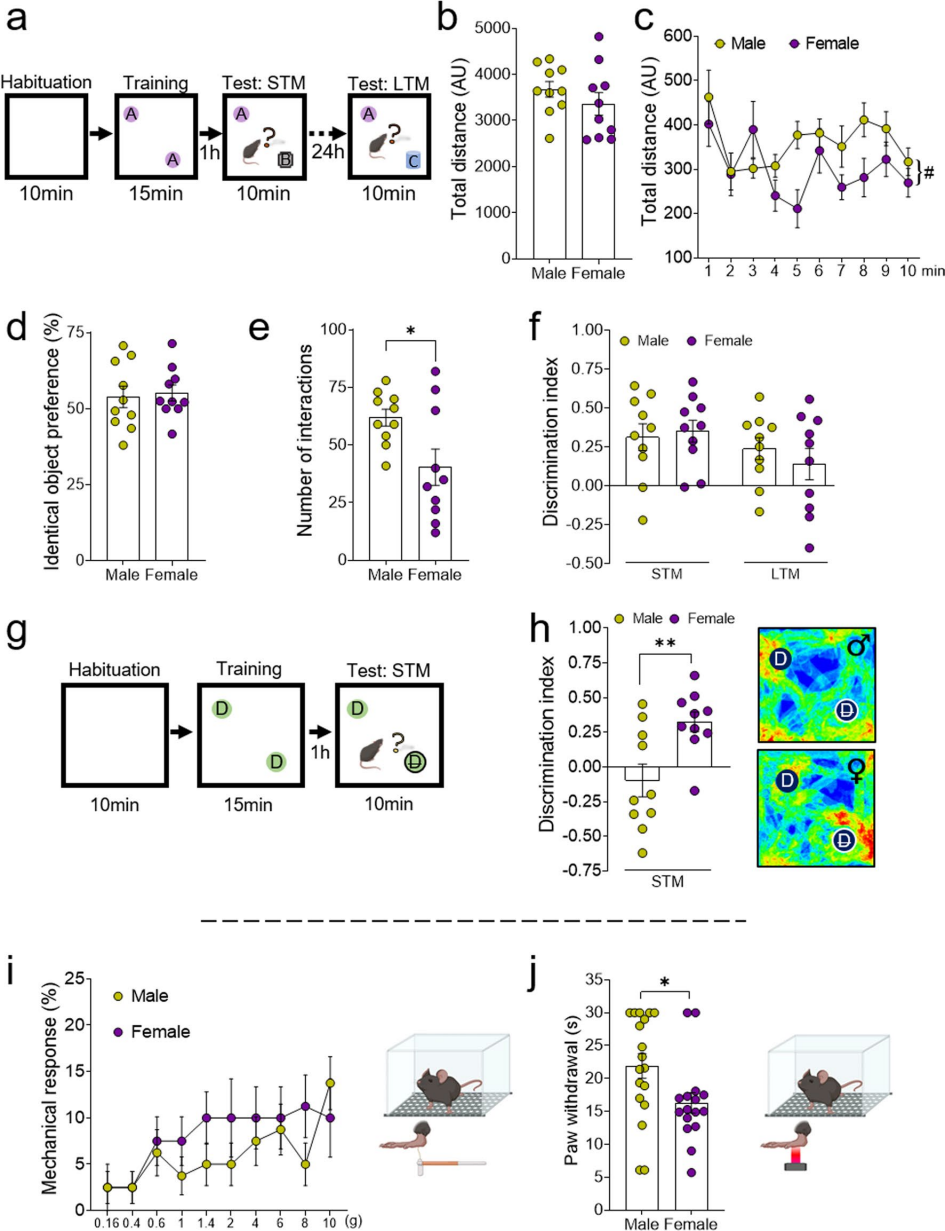
Evaluation of the NOR paradigm and the sensorial assessment of male and female mice. **a** Schematic representation of the NOR experimental design to assess short-term (STM, learning index) and long-term memory (LTM, memory index), using two different objects. Graphs depicting **b** the total distance traveled in the habituation phase of the NOR paradigm and **c** its representation in 1 min intervals. Graphs showing **d** the percentage of preference exploring identical objects (represented as A) and **e** the number of interactions during the 15-min training phase of the test. **f** Graph representing the discrimination index (DI) between objects following the STM and LTM protocols when using a novel object (represented as B in STM protocol and C in LTM protocol) that differed drastically from the familiar object. **g** Schematic representation of the NOR experimental design to assess STM using objects with minimal differences between them. **h** Graph depicting the DI following the STM protocol and representative heatmaps showing activity around the objects, when the novel object (represented as D cross out) presented strong similarity with the familiar one (represented as D). Evaluation of the **i** mechanical response in the von Frey test using calibrated filaments from 0.16 to 10 g and **j** the paw withdrawal (in seconds) in the plantar test. The data are presented as the mean ± SEM of n = 10 mice per group: *p < 0.05, **p < 0.01 vs male; #p < 0.05 vs the first minute. Females were in proestrus and estrus (P/E) stages. DI, discrimination index; AU, arbitrary units

The NOR cognitive paradigm was used to investigate whether sex might influence learning and memory, employing two different approaches in the test phase. First the novel object was not identical in shape, texture, color or size (Fig. 2a), whereas in the second approach, the novel object had minimal differences to the familiar one (Fig. 2g). Both sexes traveled a similar distance in the 10 min habituation phase (Fig. 2b, c) and the percentage of preference in exploring the two identical objects was similar in both groups (Fig. 2d), even though females interacted less frequently with the identical objects in the 15 min training phase (p < 0.05: Fig. 2e). In the test phase, where two very different objects were used, both sexes were able to distinguish the novel object and there were no significant differences between the two groups in the STM or LTM protocols (Fig. 2f). By contrast, when the novel object had minimal differences to the familiar one (Fig. 2g), only female mice were able to recognize it in the STM protocol and they achieved a higher DI than males (p < 0.01: Fig. 2h, see Table 2).

**Table 2:** Summary of statistical analysis of cognitive and sensorial behaviors

Shapiro–Wilk normality test (W, P value, Passed normality test?)										
		Male				Female				
Total distance (AU)		0.9415 0.5703 Yes				0.8977 0.2065 Yes				
Total distance (AU)		*1 min* 0.9190 0.3485 Yes		*10 min* 0.9717 0.9064 Yes		*1 min* 0.9337 0.4849 Yes		*10 min* 0.9529 0.7032 Yes		
Number of interactions		0.9701 0.8918 Yes				0.8965 0.2002 Yes				
Identical object preference (%)		0.9419 0.5738 Yes				0.9505 0.6746 Yes				
STM Discrimination index With two different objects		0.9456 0.6163 Yes				0.9461 0.6231 Yes				
LTM Discrimination index With two different objects		0.9664 0.8559 Yes				0.9433 0.5903 Yes				
STM Discrimination index With similar objects		0.9270 0.4193 Yes				0.9311 0.4589 Yes				
Nociceptive response (%)	*0.16 g*	*0.4 g*	*0.6 g*		*1 g*	*0.16 g*	*0.4 g*		*0.6 g*	*1 g*
	0.2359 < 0.0001 No	0.3512 < 0.0001 No	0,5436 < 0,0001 No		0,4330 < 0,0001 No	0,3512 < 0,0001 No	0,3512 < 0,0001 No		0,5804 < 0,0001 No	0,5804 < 0,0001 No
	*1.4 g*	*2 g*	*4 g*		*6 g*	*1.4 g*	*2 g*		*4 g*	*6 g*
	0,4954 < 0,0001 No	0,4954 < 0,0001 No	0,5804 < 0,0001 No		0,6076 < 0,0001 No	0,6265 < 0,0001 No	0,5824 < 0,0001 No		0,6710 < 0,0001 No	0,6710 < 0,0001 No
	*8 g*	*10 g*				*8 g*	*10 g*			
	0,4954 < 0,0001 No	0,6375 < 0,0001 No				0,7011 < 0,0001 No	0,5824 < 0,0001 No			
Paw withdrawal (s)		0.8804 0.0265 No				0.8522 0.0147 No				

Unpaired student’s t-test (t_(df)_)								
Total distance (AU)	t_(18)_ = 1.05							
Male total distance (AU) vs 1 min	**t**_**(18)**_ = 2.14*							
Female total distance (AU) vs 1 min	**t**_**(18)**_ = 2.21*							
Identical object preference (%)	t_(18)_ = 0.29							
Number of interactions	**t**_**(18)**_ = 2.50*							
STM discrimination index With two different objects	t_(18)_ = 0.34							
LTM discrimination index With two different objects	t_(18)_ = 0.80							
STM discrimination index With similar objects	**t**_**(18)**_ = 3.11**							

Mann–Whitney U test (U)								
Paw withdrawal (s)	U = 75.50*							

Repeated-measures ANOVA (F_(df. residual)_)								
	***Sex***	***Force (g)***	***Sex x Force (g)***					
Nociceptive response (%)	F_(1, 38)_ = 1.09	**F**_**(9, 342)**_ = 2.70**	F_(9, 342)_ = 0.72					

AU, Arbitrary Units; STM, Short-term memory; LTM, Long-term memory; df, degrees of freedom. *p < 0.05, **p < 0.01

Values in bold indicate statistically significant results

### Female mice were more sensitive to heat stimulus

To evaluate sex-dependent pain sensitivity, mechanical and heat sensory thresholds were assessed using the von Frey filament application and the plantar test on both male and female mice. No differences in mechanical sensitivity were observed between the two groups (Fig. 2i), although female mice exhibited a shorter latency in paw withdrawal than males in terms of heat nociception (p < 0.05: Fig. 2j, see Table 2).

### Structural and morphological sex differences in the mouse LC nucleus

**Fig. 3:**
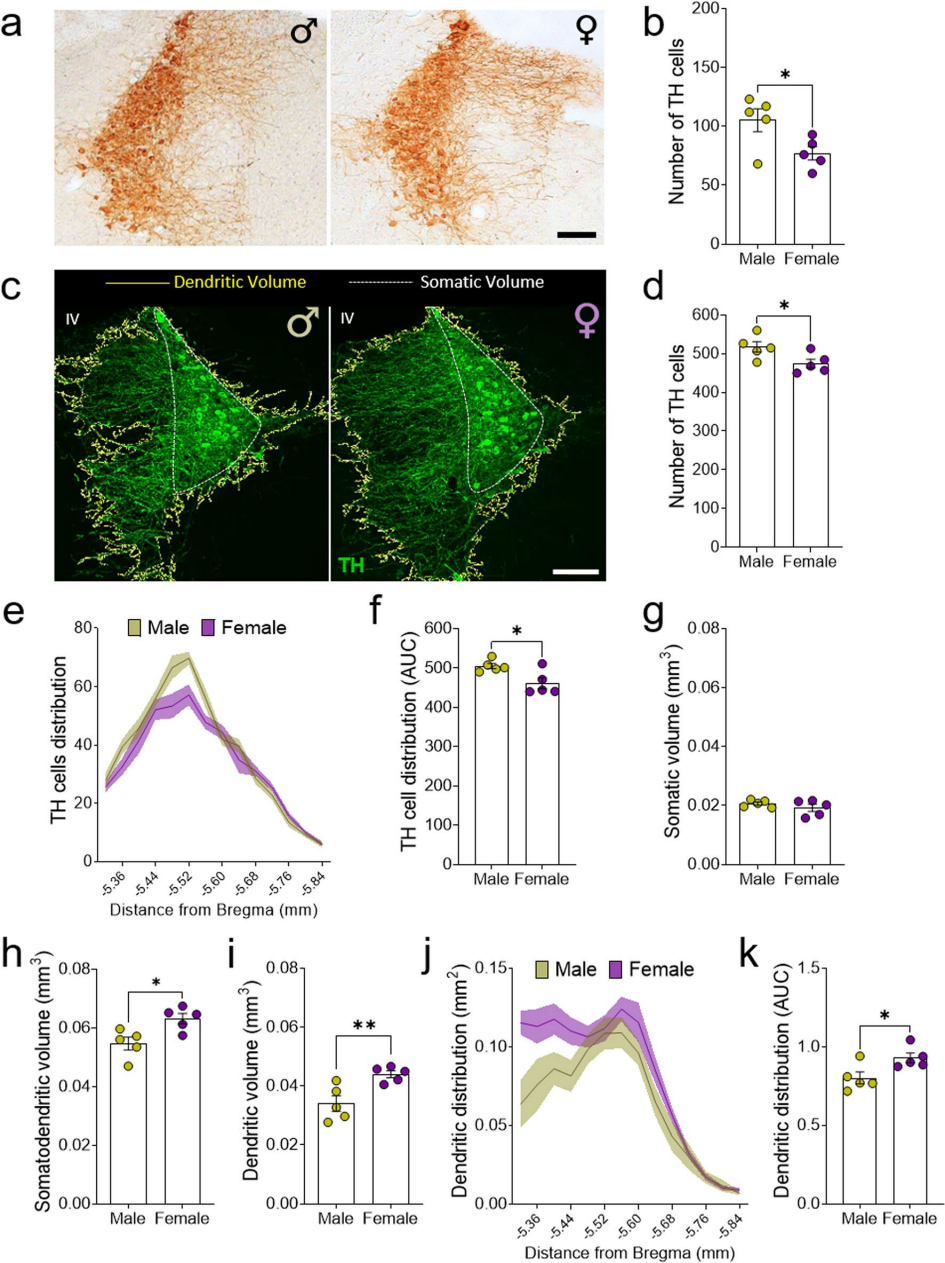
Quantification of TH^+^ cells and the LC volume in male and female mice. **a** Representative images of the LC in male and female mice stained for TH by DAB, and **b** quantification of the number of TH^+^ cells in both groups. **c** Representative confocal images of the area occupied by the soma (white line) and/or dendrites (yellow line) outlined for volume estimation. **d** Quantification of the number of TH^+^ cells detected by immunofluorescence in the entire LC. **e** Graph depicting the distribution of TH^+^ cells along the rostrocaudal axis of the LC and **f** its representation as the AUC. **g** The volume occupied by the soma, **h** somatodendrites and **i** dendrites of the entire LC. **j** Graph depicting the distribution of TH^+^ dendrites along the rostrocaudal axis of the LC and **k** its representation as the AUC. The data are presented as the mean ± SEM of n = 5 animals per group for DAB and another 5 animals per group for inmunofluorescence: *p < 0.05, **p < 0.01 vs male. Scale bars = 100 μm. IV, fourth ventricle; TH, Tyrosine Hydroxylase; DAB, 3,3′-diaminobenzidine tetrahydrochloride; AUC, area under curve

The number of TH^+^ cells in the LC was assessed by immunohistochemistry (Fig. 3a) and females had fewer TH^+^ cells in the LC than males (p < 0.05: Fig. 3b), as confirmed in immunofluorescence confocal images of the entire LC (p < 0.05: Fig. 3c, d, Additional file 2: Fig.S2a). Furthermore, along the rostrocaudal axis the difference in the number of TH^+^ in females relative to males was particularly pronounced in the central region of the LC (− 5.44 mm to − 5.60 mm from Bregma: Fig. 3e). The analysis of the AUC also showed significant differences between the two sexes in terms of the number of TH^+^ cells (p < 0.05: Fig. 3f, see Table 3).

**Table 3:** Summary of statistical analysis of LC morphology studies

Shapiro–Wilk normality test (W, P value, Passed normality test?)		
	Male	Female
Number of TH cells (DAB)	0.8148 0.1065 Yes	0.9918 0.9856 Yes
Number of TH cells (IF)	0.9817 0.9433 Yes	0.9283 0.5851 Yes
AUC of TH cells distribution	0.9090 0.4614 Yes	0.7954 0.0743 Yes
Somatic volume (mm^3^)	0.9421 0.6806 Yes	0.8673 0.2556 Yes
Somatodendritic volume (mm^3^)	0.9288 0.5882 Yes	0.9480 0.7231 Yes
Dendritic volume (mm^3^)	0.9440 0.6947 Yes	0.9195 0.5268 Yes
AUC of Somatic distribution	0.9382 0.6529 Yes	0.8328 0.1460 Yes
AUC of Somatodendritic distribution	0.8018 0.0838 Yes	0.9155 0.5016 Yes
AUC of Dendritic distribution	0.7903 0.0673 Yes	0.8311 0.1417 Yes

Unpaired Student’s t-test (t_(df)_)		
Number of TH cells (DAB)	**t**_**(8)**_ = 2.51*	
Number of TH cells (IF)	**t**_**(8)**_ = 2.44*	
AUC of TH cells distribution	**t**_**(8)**_ = 2.89*	
Somatic volume (mm^3^)	t_(8)_ = 1.08	
Somatodendritic volume (mm^3^)	**t**_**(8)**_ = 3.06*	
Dendritic volume (mm^3^)	**t**_**(8)**_ = 3.47**	
AUC of Somatic distribution	t_(8)_ = 0.91	
AUC of Somatodendritic distribution	t_(8)_ = 1.61	
AUC of Dendritic distribution	**t**_**(8)**_ = 2.61*	

TH, Tyrosine hydroxylase; DAB, 3,3′-diaminobenzidine tetrahydrochloride; IF, immunofluorescence; AUC, area under the curve; df, degrees of freedom

*p < 0.05; **p < 0.01

Values in bold indicate statistically significant results

According to the Cavalieri’s principle, the analysis of the volume occupied by noradrenergic somas did not show differences between the groups (Fig. 3g), although female mice had a significantly higher somatodendritic volume than males (p < 0.05: Fig. 3h), as a result of a higher dendritic volume in the female LC relative to that of males (p < 0.01: Fig. 3i). The somatic (Additional file 2: Fig. S2b, c) and somatodendritic (Additional file 2: Fig. S2d, e) distribution in the rostrocaudal axis was subsequently analyzed by plotting the somas and the somatodendritic occupied areas in each LC section, which failed to reveal any significant differences. However, when the dendritic distribution in the rostrocaudal axis was analyzed by plotting the area occupied by dendrites in each LC section, a larger area of the LC was occupied by dendrites in females, particularly in the rostral region of the LC nucleus (− 5.32 mm to − 5.44 mm from Bregma: Fig. 3j). The AUC when the LC dendrite distribution in the LC was plotted also revealed significant differences between the sexes (p < 0.05: Fig. 3k, see Table 3).

### The passive properties and excitability of LC neurons differ between male and female mice

**Fig. 4:**
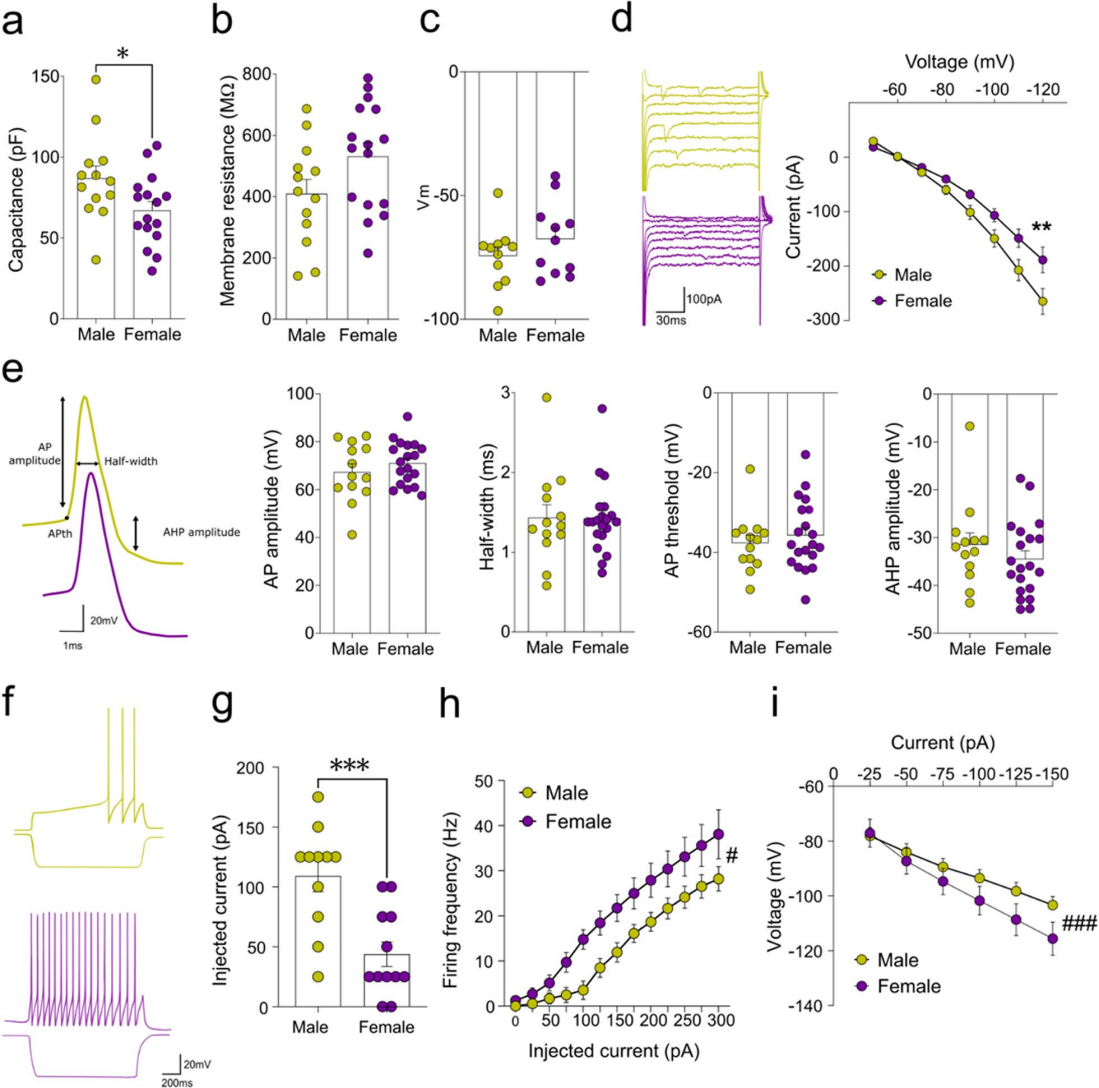
Evaluation of the passive properties and excitability of LC neurons in male and female mice. Population graphs depicting differences in **a** the membrane capacitance, **b** a slight difference in the membrane resistance and **c** similar membrane resting potentials. **d** Representative example and graph of the IRK currents. **e** Representative example of the action potential (AP) and corresponding parameters, such as the amplitude, half-width, threshold and after hyperpolarization potential (AHP). **f** Representative examples of the voltage responses of identified LC neurons to current injection of + 100 and − 100 pA, respectively. **g** Rheobase. **h** Graph showing the activity driven in response to the injection of positive currents (+ 25 pA steps). **i** Graph showing the voltage deflections in response to the injection of negative currents (− 25 pA steps). The data are presented as the mean ± SEM of n = 5 mice per group: *p < 0.05, **p < 0.01 ***p < 0.001 vs male. ANOVA (sex factor): ^#^p < 0.05, ^###^p < 0.001

Since we found behavioral and structural differences in the LC, we further studied if the mice sex conditions the electrical activity of LC neurons. We used whole-cell patch clamp recordings to evaluate the intrinsic properties and excitability of LC neurons, identifying a smaller membrane capacitance (Cm, p < 0.05: Fig. 4a) and a tendency towards a higher membrane resistance (Rm, p = 0.07: Fig. 4b) in female animals, yet with a similar membrane resting potential to males (Fig. 4c). IRK currents were smaller in female animals (p < 0.01: Fig. 4d), reflecting possible differences in cell excitability. Nevertheless, several parameters related to the action potential (AP) were similar between male and female mice, such as amplitude, half-width, threshold and after hyperpolarization amplitude (AHP: Fig. 4e). In addition to the changes in the passive properties, excitability was enhanced in the female mice (Fig. 4f), which showed a smaller rheobase (p < 0.001: Fig. 4g), together with a faster firing frequency (p < 0.05: Fig. 4h) and a larger voltage-deflection in response to positive or negative current injection (25 pA steps, p < 0.001, ANOVA: Fig. 4i, see Table 4).

**Table 4:** Summary of statistical analysis of the patch clamp study

Shapiro–Wilk normality test (W, P value, Passed normality test?)			
	Male	Female	
Capacitance (pF)	0.9381 0.4329 Yes	0.9762 0.9262 Yes	
Resistance (MΩ)	0.9726 0.9235 Yes	0.9438 0.3980 Yes	
Resting potential (Vm)	0.8819 0.0757 Yes	0.9299 0.1539 Yes	
Inward-rectifier potassium channels (IRK)	0.9469 0.6795 Yes	0.9563 0.7742 Yes	
AP amplitude (mV)	0.9378 0.4288 Yes	0.9596 0.5650 Yes	
Half-width (ms)	0.8983 0.1268 Yes	0.8688 0.0112 No	
Threshold (mV)	0.8960 0.1177 Yes	0.9664 0.6768 Yes	
AHP amplitude (mV)	0.8480 0.0269 No	0.9406 0.2461 Yes	
Injected current (pA)	0.8698 0.0650 Yes	0.9132 0.2659 Yes	
Firing frequency (Hz)	0.8979 0.1253 Yes	0.9389 0.4426 Yes	
Voltage (mV)	0.9885 0.9851 Yes	0.9860 0.9770 Yes	

Unpaired Student’s t-test (t_(df)_)			
Capacitance (pF)	**t**_**(27)**_ = 2.19*		
Resistance (MΩ)	**t**_**(27)**_** = 1.90**		
Resting potential (Vm)	t_(31)_ = 1.16		
AP amplitude (mV)	t_(30)_ = 0.98		
Threshold (mV)	t_(31)_ = 0.66		
Injected current (pA)	**t**_**(21)**_ = 3.95***		

Mann–Whitney U test (U)			
Half-width (ms)	U = 120.5		
AHP amplitude (mV)	U = 108		

Repeated-measures ANOVA (F_(df. residual)_)			
	*Sex*	*Current*	*Sex x Current*
Inward-rectifier potassium channels (IRK)	**F**_**(1, 19)**_ = 4.96*	**F**_**(7,133)**_ = 178.09***	**F**_**(7,133)**_ = 5.06***
Firing frequency (Hz)	**F**_**(1, 16)**_ = 5.06*	**F**_**(12,192)**_ = 78.80***	**F**_**(12,192)**_ = 2.17*
Voltage (mV)	F_(1, 19)_ = 1.09	**F**_**(5, 95)**_ = 180.26***	**F**_**(5, 95)**_ = 7.86***

df, degrees of freedom

*p < 0.05; ***p < 0.001

Values in bold indicate statistically significant results

## Discussion

Through immunohistochemistry studies, sex differences in the noradrenergic LC nucleus of C57BL/6J mice were demonstrated here. This was reflected by a relatively smaller number of TH^+^ cells in the central region of the female LC and a larger volume of the rostral female LC occupied by dendrites. Furthermore, whole-cell patch-clamp electrophysiology revealed that LC neurons from female mice had distinct intrinsic properties and cell excitability, summarized by a smaller membrane capacitance and enhanced excitability. In behavioral studies female mice exhibit more anxiety-related behaviors, although females and males developed similar depressive-like behaviors. Females outperformed males in memory tasks that involved distinguishing between objects with minor differences and they also exhibited greater thermal pain sensitivity, yet no differences were found when mechanical stimuli were applied.

We used different immunohistochemistry approaches to compare the number of TH^+^ cells along the rostrocaudal axis of the LC nucleus in female and male mice. DAB and immunofluorescence studies indicated that there were fewer TH^+^ cells in the LC of females, particularly in the central region of the LC. Previous studies demonstrated sex differences in LC neurons, mainly in rats, and strain and age-dependent results were reported. While there were more neurons in the LC of female Wistar rats compared to males [48, 49], this difference was not evident in Long-Evans rats [50, 51]. Although there is little data from mice, no differences in TH^+^ cells were found in the LC of adult female and male C57BL/6J mice [52]. However, we found here more TH^+^ cells in the male LC and a higher proportion of TH^+^ cells mainly in the central region of the LC. The differences between these two studies could be due to the methodological approaches, as the earlier study counted the number of TH^+^ cells in 3 serial sections spanning the rostral to caudal extent of the LC, while we counted the number of TH^+^ cells by immunofluorescence in all sections covering the entire LC region. Taking into account that the main differences were found in the central region of the male LC, it is plausible that data collection from serial LC sections could mask differences along the rostrocaudal axis.

Although there were fewer TH^+^ cells in the female mice LC, there were no differences in the volume occupied by their somas between females and males. Interestingly, when the volume occupied by TH^+^ soma or dendrites was analyzed separately, a higher dendritic volume was evident in the female LC, predominantly in the rostral LC. Studies in Sprague–Dawley rats indicated that the dendritic arbor of the LC is more complex and extends further in female than in male rats [25]. Moreover, synaptophysin expression was stronger in the LC of female rats, which might suggest more synaptic inputs in this region. Interestingly, hypothalamic-projecting LC neurons are located rostrally and consequently, the rostral LC presumably receives more corticotropin releasing factor (CRF), which has been reported to activate LC neurons and promote an anxiety-like behavioral phenotype [13].

LC cells were evaluated functionally by whole-cell patch-clamp electrophysiology, identifying a lower membrane capacitance and slightly higher resistance in the LC of female mice. Despite these differences, the properties of the action potential were similar in male and female mice, suggesting similar implications of ion channels in shaping the action potential [53]. Interestingly, in female mice higher excitability was seen than in males, as corroborated further through a lower rheobase and smaller IRK currents. Previous studies on C57BL/6 background mice also found enhanced LC excitability in females relative to males [54]. Females were also more sensitive to hyperpolarizing stimuli than males, suggesting that female LC cells are more excitable and functionally more sensitive to external inputs. This also agrees with previous electrophysiological findings in anesthetized animals showing that female LC neurons fire faster than those of males when exposed to hypotensive stress [55]. Importantly, our electrophysiological assessment does not make it possible to demonstrate the extensive connectivity of the LC or the consequences of the afferents it receives for the modelling of cellular activities. Indeed, it is currently known that the LC has a heterogeneous organization and function with discrete modes of activation, whereby different modules of noradrenergic neurons enter segregated operational modes [56]. Therefore, future electrophysiological experiments in combination with neural tracers of specific subpopulations are still pending.

The activity of LC-noradrenergic neurons is required to elicit acute stress-induced anxiety and indeed, optogenetic/chemogenetic activation of LC neurons is itself anxiogenic [57, 58]. Thus, sex differences in the LC might be involved in the behavioral differences found between sexes. The evaluation of anxiety-like behavior through the EPM, the light/dark and the OFT paradigms are based on unconditioned reactions, consistently showing that female mice have a higher index of anxiety. These sex differences are in agreement with reports [59] but not with others [60]. The discrepancy in the data obtained from different studies may be masked by factors that influence these unconditioned anxiety tests, such as locomotion [61]. Thus, studies reporting no differences in the time spent in the open side, classically found that female mice showed significantly higher scores in distance moved or time spent walking parameters [60] introducing a clear interpretation bias regarding anxiety state. Our results did not show sex differences in the motor activity, providing greater confidence about the anxiogenic state of females. The higher anxiety found in female mice could also be linked to the estrous cycle because estrogen up-regulates TH gene transcription [62] and mRNA expression in the LC [63, 64], as well as the noradrenaline release in multiple brain areas [65, 66]. Thus, it is likely that stronger reactivity of the LC is due to higher estrogens levels promoting an anxiogenic phenotype. In line with the anxiety assessment, we also found that females were less able to remove pellets from the burrow than males, and that they had a lower latency to groom than males, although their total grooming time was similar in the splash test. These are measurements of well-being and self-care behaviors [67] in which males outperformed females. Accordingly, other studies reported that less time is spent burrowing by female mice than males [68] and that the burrow formed by males is longer than that of females [69]. Thus, males and females perform burrowing behavior differently which may reflect differences in basal well-being.

Interestingly, males and females showed similar behavioral despair in the TST and FST tests, although adopting different active behaviors, such as females climbing more in the TST. Climbing behavior in cages is considered stereotypic-like behavior, suggesting that increased climbing by animals may reflect psychological distress and anxiety [70]. Earlier data from rodents showed females routinely adopt more “grid-climbing” activity in cages than males [71], which could be related to greater curiosity as part of the exploratory behavior of unknown environments outside the cage. Despite no sexual differences were found in response to a situation of short-term inescapable stress like the TST or FST, further studies using other stress modalities of longer duration, such as the repeated social defeat paradigm [72], where the LC might be involved, would be of great interest.

The enhanced structural and functional LC sensitivity in female rodents might also be involved in learning and memory in the NOR paradigm [73–75]. To test STM as a learning index and LTM as an index of memory, we adopted the NOR cognitive paradigm. We found that both males and females had similar baseline preferences for the two identical objects presented in the training phase. However, females required less interactions with identical objects to display the same preference as males, suggesting that they are more efficient in this recognition. In addition, when a novel object was presented in the test phase, the learning and memory index of males and females was similar in terms of recognition. Interestingly, when an object with minimal differences to the familiar one was presented in a STM protocol, females exhibited a higher DI. While advantages in the recognition of novel objects have been reported in female Long Evans rats [76], this is not so clear in female C57BL/6J mice. Indeed, male preference was reported for exploring a novel object [77], yet a clear advantage of females over males in the recognition of a novel object has been found [78]. Here, we reported similar preferences for a novel object in STM and LTM protocols using two very different objects, although females had a clear advantage in recognizing minimal differences between objects. These results are consistent with previous findings from humans, whereby object details went unnoticed by men but women were more adept at distinguishing such details [78–81]. Presentation of a novel stimulus can trigger an increase in corticosterone plasma levels, an index of stress [82]. Thus, the precise encoding of visual details in females could be related to their capacity to detect emotional or stressful events rapidly, as females are generally more emotional and stressed than males [83–85]. All these results align with the existing literature on mice and other species, highlighting the importance of controlling object characteristics when performing this type of task and encourage the evaluation of different learning modalities [60, 86].

We also found that females displayed greater thermal sensitivity in the plantar test, although no differences were found in the response when applying a mechanical stimulus. At least some important aspects of pain processing are robustly sex-dependent [87, 88] and although further research is necessary, most studies in rodents show females to be more sensitive to pain stimuli [87–89]. In addition, studies in humans show that women are more sensitive to pain than men, evident through greater sensitivity to the exposure for first time to a thermal stimulus [7, 90–92]. However, studies that employed mechanical stimuli reported lower pain thresholds for women but also found no sex differences [93–95]. Although there is evidence that LC projections are involved in thermal thresholds in male rodents [96, 97], to date this issue has not been addressed in females.

With the increasing interest in exploring sexual differences in rodent behaviors, some laboratories are reporting sexual differences that others do not find. Many factors may explain such inconsistencies, such as strain, estrous cycle, and animal age [59, 98, 99]. Furthermore, the environment is also known to play a crucial role, including factors like housing temperature, light intensity, handling and even the sex of the experimenter [100–104]. Therefore, to determine the impact of sex on rodent behaviors related to emotions, cognition, and pain, systematic reviews or meta-analyses are mandatory, like some already available [1, 3, 88]. However, our study holds the value of exploring rodent behavior in parallel with LC physiology, in a specific mice strain in our laboratory conditions. Having this in mind, we found that female mice have a higher anxiogenic profile, yet they better distinguish minimal differences in objects and have a lower thermal pain threshold than males. However, no differences were detected when assessing behavioral despair and mechanical responses. In parallel, the dendritic volume in the LC is greater in female mice, especially in regions receiving inputs from areas that process salient information. LC neurons in female mice differ in their intrinsic properties and they are more excitable than in males, which may contribute to the observed behavioral differences. Although causal experiments remain pending, and considering that the LC may act as a mediator of emotions, cognition and pain in response to stimuli, as well as dynamic environmental challenges, these structural and functional sex differences observed in the LC may lead to heightened emotional arousal in females. This arousal may be adaptive but it may also contribute to higher rates of stress-related psychiatric disorders in women, such as post-traumatic stress disorder or generalized anxiety. Based on all of these findings, it is clear that further studies are needed to decipher and understand the sexual differences in LC-related behaviors.

## Perspectives and significance

Our data demonstrated pronounced sex differences in the LC nucleus and in the LC-related behavior. Here, females reported fewer noradrenergic cells and a larger volume of dendrites in the rostral region of the LC. Also, LC electrophysiology studies revealed that female mice showed an enhanced cell excitability. This higher excitability might explain why females are more sensitive to relevant sensorial inputs as shown by better performance recognizing minimal differences between objects and higher anxiety-related behaviors than males. Overall suggest the importance of knowledge of sex differences in the CNS to understand brain and related pathologies in a sex-specific manner.

### Supplementary Information

**Additional file 1: Figure S1. a** Representative images showing the different stages of the estrous cycle in female mice. Proestrus is characterized by nucleated epithelial cells, estrus by cornified epithelial cells, and metestrus and diestrus by the presence of leukocytes. The results of anxiety-like behavior expressed as **b** the percentage of time spent in the open arms of the EPM for males and females, represented by estrous cycle stages. Graphs depicting (**c**) the total activity in arbitrary units (AU) and **d** the number of entries into the open arms in the EPM. **e** The latency to enter the dark compartment (in seconds) and **f** the number of transitions between compartments in the light/dark test. **g** Graph showing the total distance traveled in the OFT. **h** Graph depicting the latency to grooming (in seconds) in the splash test, **i** the percentage of animals that perform clasping, curling, swinging and climbing behavior in the TST, and **j** the latency (in seconds) to immobility in the FST. Females were in proestrus and estrus (P/E) stages in (**e**) to (**j**) behavioral tests. The data are presented as the mean ± SEM of n = 7–10 mice per group: **p < 0.01, ***p < 0.001 vs male; #p < 0.05 vs female P/E. P/E, Proestrus/Estrus; D/M, Diestrus/Metestrus. Scale bar = 50 µm.

**Additional file 2: Figure S2. a** Representative immunofluorescence confocal images of the entire LC region along the rostrocaudal axis (distances from Bregma in mm). **b** Graph depicting the distribution of the area occupied by the soma in the rostrocaudal axis of the LC, followed by **c** the AUC analysis. **d** Graph depicting the distribution of the area occupied by the somatodendritic region along the rostrocaudal axis of the LC, followed by **e** AUC analysis. The data are presented as the mean ± SEM of n = 5 animals per group. Females were in proestrus and estrus (P/E) stages. Scale bar = 100 μm. TH, Tyrosine Hydroxylase; AUC, area under the curve.

## Data Availability

The datasets used and/or analysed during the current study are available from the corresponding author on reasonable request.
