# Supplementary figures and images for: Sexual differences in locus coeruleus neurons and related behavior in C57BL/6J mice
Source: Biol Sex Differ. 2023 Sep 28;14:64. doi: 10.1186/s13293-023-00550-7 (PMC10540344; doi:10.1186/s13293-023-00550-7)

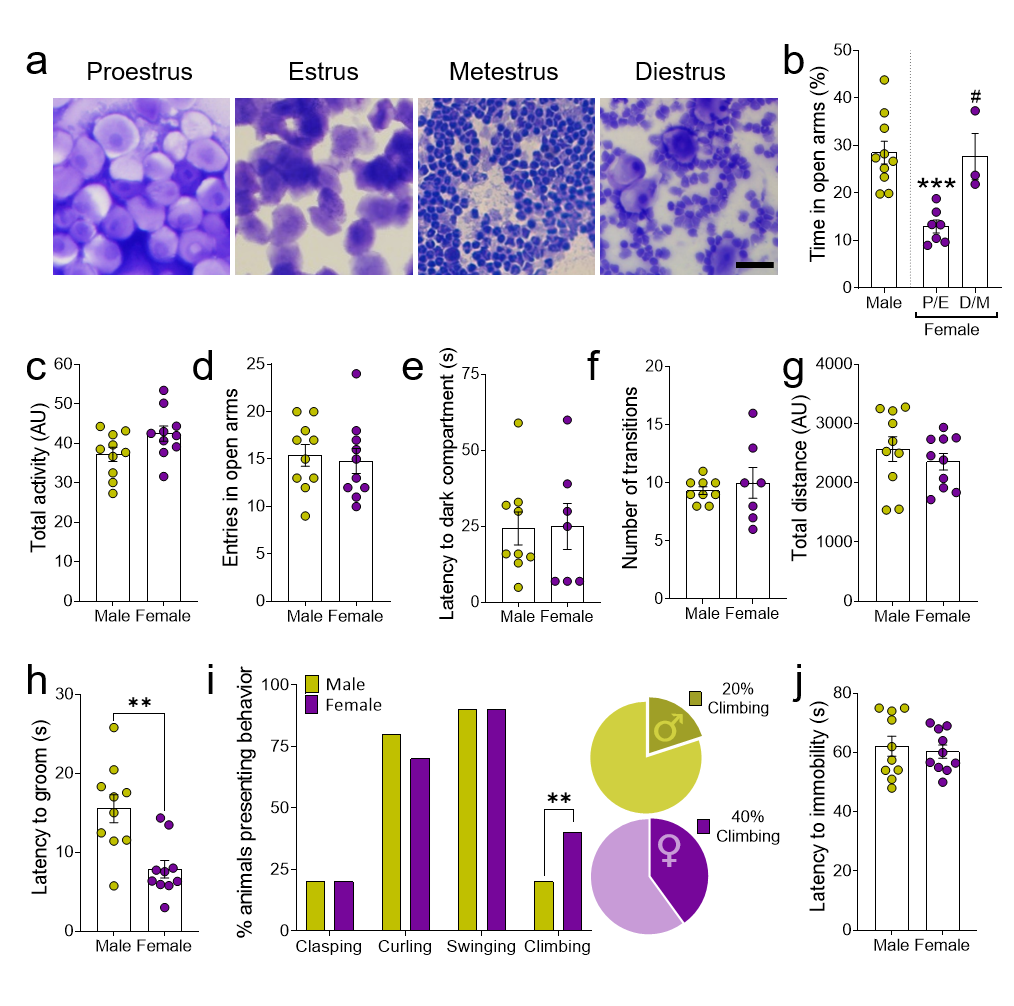

Supplement: Supplementary file 1 — Additional file 1: Figure S1. a Representative images showing the different stages of the estrous cycle in female mice. Proestrus is characterized by nucleated epithelial cells, estrus by cornified epithelial cells, and metestrus and diestrus by the presence of leukocytes. The results of anxiety-like behavior expressed as b the percentage of time spent in the open arms of the EPM for males and females, represented by estrous cycle stages. Graphs depicting (c) the total activity in arbitrary units (AU) and d the number of entries into the open arms in the EPM. e The latency to enter the dark compartment (in seconds) and f the number of transitions between compartments in the light/dark test. g Graph showing the total distance traveled in the OFT. h Graph depicting the latency to grooming (in seconds) in the splash test, i the percentage of animals that perform clasping, curling, swinging and climbing behavior in the TST, and j the latency (in seconds) to immobility in the FST. Females were in proestrus and estrus (P/E) stages in (e) to (j) behavioral tests. The data are presented as the mean ± SEM of n = 7–10 mice per group: **p < 0.01, ***p < 0.001 vs male; #p < 0.05 vs female P/E. P/E, Proestrus/Estrus; D/M, Diestrus/Metestrus. Scale bar = 50 µm. [file 13293_2023_550_MOESM1_ESM.tif]

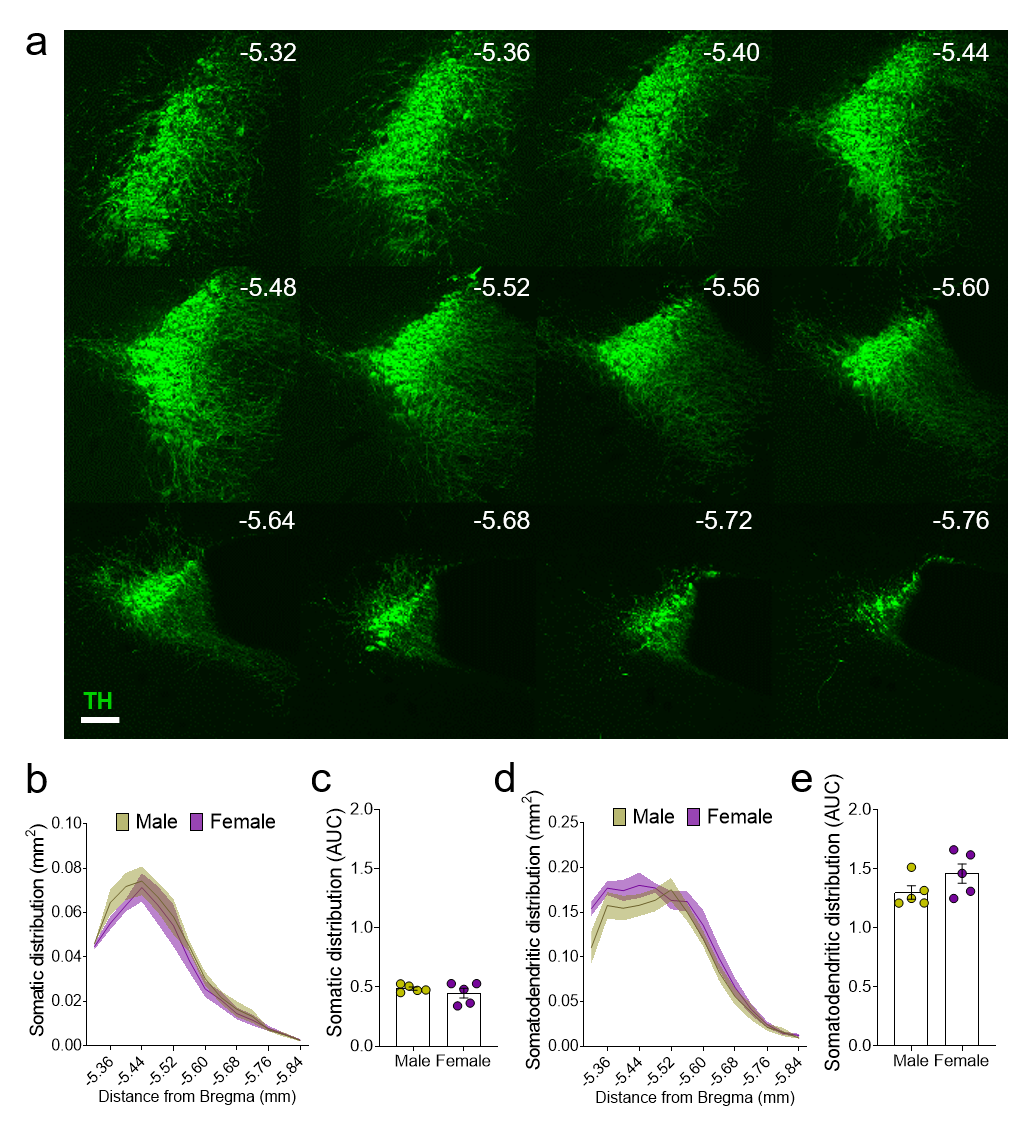

Supplement: Supplementary file 2 — Additional file 2: Figure S2. a Representative immunofluorescence confocal images of the entire LC region along the rostrocaudal axis (distances from Bregma in mm). b Graph depicting the distribution of the area occupied by the soma in the rostrocaudal axis of the LC, followed by c the AUC analysis. d Graph depicting the distribution of the area occupied by the somatodendritic region along the rostrocaudal axis of the LC, followed by e AUC analysis. The data are presented as the mean ± SEM of n = 5 animals per group. Females were in proestrus and estrus (P/E) stages. Scale bar = 100 μm. TH, Tyrosine Hydroxylase; AUC, area under the curve. [file 13293_2023_550_MOESM2_ESM.tif]
